# Comprehensive Analysis of Leukocytes, Vascularization and Matrix Metalloproteinases in Human Menstrual Xenograft Model

**DOI:** 10.1371/journal.pone.0016840

**Published:** 2011-02-17

**Authors:** Yong Guo, Bin He, Xiangbo Xu, Jiedong Wang

**Affiliations:** 1 Graduate School, Peking Union Medical College, Beijing, People's Republic of China; 2 Department of Cell Biology, National Research Institute for Family Planning, Beijing, People's Republic of China; State Key Laboratory of Reproductive Biology, Institute of Zoology, Chinese Academy of Sciences, China

## Abstract

In our previous study, menstrual-like changes in mouse were provoked through the pharmacologic withdrawal of progesterone with mifepristone following induction of decidualization. However, mouse is not a natural menstruation animal, and the menstruation model using external stimuli may not truly reflect the occurrence and development of the human menstrual process. Therefore, we established a model of menstruation based on human endometrial xenotransplantation. In this model, human endometrial tissues were transplanted subcutaneously into SCID mice that were ovarectomized and supplemented with estrogen and progestogen by silastic implants with a scheme imitating the endocrinological milieu of human menstrual cycle. Morphology, hormone levels, and expression of vimentin and cytokeratin markers were evaluated to confirm the menstrual-like changes in this model. With 28 days of hormone treatment, transplanted human endometrium survived and underwent proliferation, differentiation and disintegration, similar to human endometrium in vivo. Human CD45^+^ cells showed a peak of increase 28 days post-transplantation. Three days after progesterone withdrawal, mouse CD45^+^ cells increased rapidly in number and were significantly greater than human CD45^+^ cell counts. Mouse CD31^+^ blood vascular-like structures were detected in both transplanted and host tissues. After progesterone withdrawal, the expression levels of matrix metalloproteinases (MMP) 1, 2, and 9 were increased. In summary, we successfully established a human endometrial xenotransplantation model in SCID mice, based on the results of menstrual-like changes in which MMP-1, 2 and 9 are involved. We showed that leukocytes are originated from in situ proliferation in human xenografts and involved in the occurrence of menstruation. This model will help to further understand the occurrence, growth, and differentiation of the endometrium and the underlying mechanisms of menstruation.

## Introduction

The study of mechanisms of menstruation has been hampered by the lack of a suitable experimental model. In nature, only humans, a few Old World primates and other mammals, such as bats, elephants and shrews, have menstrual cycles [1]. Thus, there are few animals that can be used to study the menstrual cycle. Currently, non-human primates are the best candidate animals and the most often used for menstruation model, but such studies have been limited by many considerations, including sources, cost and ethics. Since the 1980's, our and other laboratories have established a model for menstruation in mouse, which does not menstruate naturally, using artificially induced decidualization to achieve uterine menstrual-like changes [2]–[4]. Establishment of a mouse menstrual-cycle model by providing hormones artificially has provided a more convenient research platform for the study of human menstruation. However, mouse is not a naturally menstruating animal, and the mouse model does not truly reflect the occurrence and development of the human menstrual process. Researchers have also isolated and co-cultured human endometrial cells in vitro [5], but these in vitro models are limited in the understanding of mechanisms of menstruation due to lack of tissue organization, blood supply, and an endocrine-regulated microenvironment. Taken together, an ideal model should maintain endometrial tissue integrity without losing the physiological characteristics of such a microenvironment.

Previous studies conducted in nude mice showed that the harvest rates of transplanted human endometrial cells were quite low [6]–[9]. Severe combined immunodeficiency (SCID) mice, first successfully established in 1983 by Bosma *et al*. [10], showed a negative response in tests of T and B lymphocyte function, with no cellular or hormonal immune response to exogenous antigen and a high survival rate of transplants. Therefore, they have become a unique animal model for immunology research. In 2006, Ozawa *et al*. [11] established a human uterus model of endometriosis by transplanting endometrial tissues into SCID mice, which suggested that human endometrial xenografts could survive and grow in SCID mice.

In this study, we aimed to establish a human menstrual xenotransplantation model in SCID mice by transplanting human endometrial tissues, and treating the mice with estrogen and progestogen to mimic the hormonal changes in human menstrual cycle. Changes of endometrial morphology and structure, as well as the expression of prolactin (PRL), a decidual surface marker, and matrix metalloproteinases (MMPs) were examined at different time points. We also analyzed leukocyte infiltration and angiogenesis in the model.

## Results

### The fate of human endometrial xenografts

To reduce the variations, all the samples were taken at the proliferative phase of menstrual cycle in the experiments. A total of 45 mice were used in the study, of which 5 mice died before the time point of histological analysis. 26 mice contained human endometrial-like tissues were examined by histological analysis of tissue sections at different indicated time points. A detailed statistical analysis for recovery rates of the transplanted tissues is shown in **Table 1**. With support of hormones, about 10∼55% of transplanted tissues remain intact in our study. Most of the harvested endometrial-like tissues showed similar changes in each group.

**Table 1:** Recovery rates of the human endometrial xenografts.

Group	n a	Recovery rates b (%) (range)
Control	5	24.2±10.6 (12.5∼37.5)
14d	4	25.5±5.2 (18∼30)
21d	4	26.3±5.7 (18∼30)
28d	7	29.3±16.9 (10∼55)
31d	6	31.4±14.2(12.5∼50)

a Number of mice in which endometrial-like tissues were detected by histological analysis of tissue sections.

b Number of harvested endometrial-like tissues/Number of transplanted tissues.

**Figure 1:**
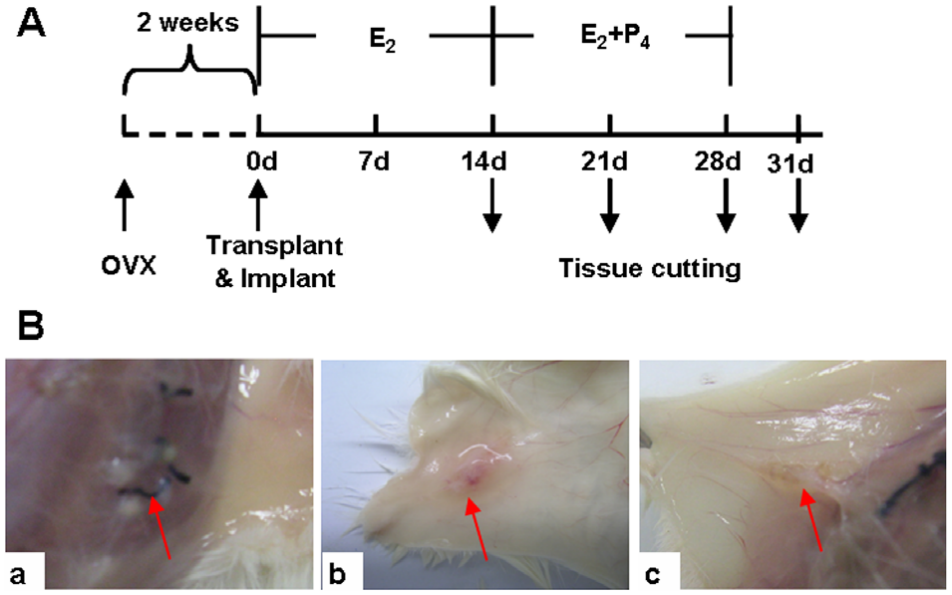
Morphological characteristics of xenotransplanted endometrial tissues. (**A**) Scheme and grouping of the experiment. The ovarectomized mice were allowed to recover for 2 weeks in order to get endogenous hormones free. E_2_ was administrated in the whole process while P_4_ was given during day 14 to day 28. The transplanted endometrium tissues were harvested at different time points indicated by downward arrowheads. (**B**) Photos of transplanted endometrial tissues. a: 28d group; b: 31d group; c: control group without hormone treatment. The red arrowheads indicated transplanted tissues. OVX, removal of both ovaries; E_2_, estradiol; P_4_, progesterone.

The transplanted tissues showed significant differences in general morphology (indicated by red arrows in **Fig. 1B**) between the different groups. 28 days after hormone treatment (28d group), the tissue fragments with a diameter of 2–3 mm were visible in the subcutaneous tissues (**Fig. 1B** **a**). The size of the tissue fragments was increased 1–2 fold compared with that before transplanted, and most tissue fragments were white in color. Blood vessels were clearly visible in the surrounding tissues. Some tissues in the withdrawal group (31d group) were blood red in color (**Fig. 1B b**), indicating that a good blood supply was established. Compared with those in 28d and 31d group, the tissues transplanted in the control group without hormone support were significantly smaller in size, and wax yellow in color (**Fig. 1B c**), which suggested that these tissues either stopped growing completely or had their growth inhibited.

### Histological examination

**Figure 2:**
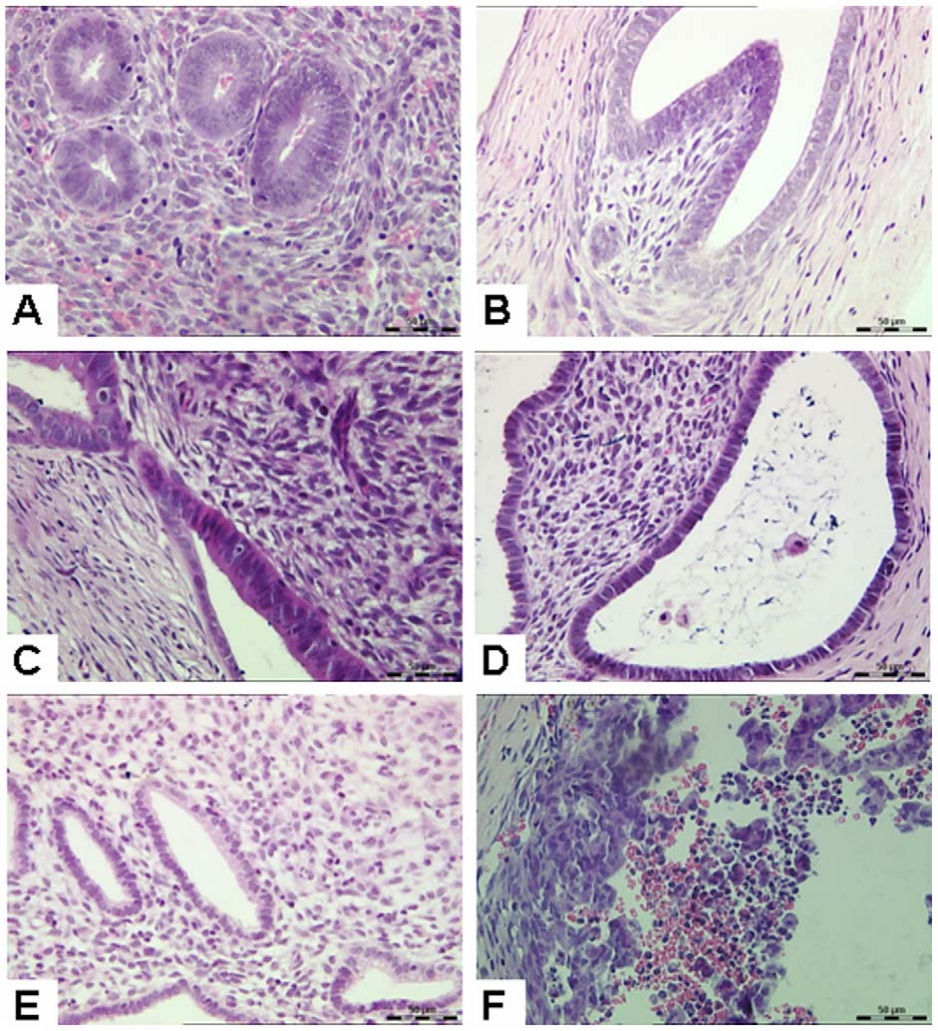
Histological examination of xenotransplanted human endometrial tissues. (**A**) Human endometrial tissues before transplantation. Endometrial tissues showed simple columnar epithelium and dense stroma, which characterized a typical human early proliferative phase endometrium. (**B**) Control group without hormone treatment. The tissue fragments were small in size and increased in lumen diameter. (**C**) 14d group (E_2_ provided alone). Glandular epithelium was in high columnar form with a significant pseudostratification of the nuclei. (**D**) 21d group (E_2_ provided for 21 days of which P_4_ provided for last 7 days). Glandular epithelial cells were changed into low columnar. Subnuclear vacuolation was clearly visible in the glandular epithelial cells with nuclei close to the basilar membrane, whereas stromal cell density was decreased. (**E**) 28d group (E_2_ provided for 28 days of which P_4_ provided for last 14 days). The cell-cavity surface with irregular margins contained a large number of small secretion bubbles. Interstitial edema was observed, stromal cells were enlarged, and the nuclei clearly showed a typical decidual-like stromal change. (**F**) 31d group (hormones were provided for 28 days and then no hormone support for the remaining 3 days). A large number of leukocytes were infiltrated, and the endometrial tissue structure was disintegrated with erythrocyte leakage. Original magnification: 400× (H&E).

Before transplantation, endometrial tissues showed simple columnar epithelium, small glandular lumen, and dense stromal cells with most of them being spindle-like, which characterized a typical human early proliferative phase endometrium (**Fig. 2A**). After transplantation and hormone treatment, the endometrial tissue fragments increased in volume and were surrounded by a connective tissue capsule with a clear boundary. In the absence of hormones, the tissue fragments of the control group did not grow, and were significantly smaller than those of the hormone-treated groups, although the lumen was enlarged (**Fig. 2B**). After 14 days of 17β-estradiol (17β-E_2_) treatment (14d group), the following features were shown: tissue volume increased significantly; glandular epithelium was in high columnar form with a significant pseudostratification of the nuclei; glandular lumen was expanded, stromal cells were mostly spindle or round in shape; the cell cytoplasm was scarce (**Fig. 2C**). In the 21d group, the glandular lumen was expanded more, with cavities contained secretions and exfoliated epithelial cells in the cavity. Glandular epithelial cells were changed into low columnar in appearance. Subnuclear vacuolation was clearly visible in the glandular epithelial cells with nuclei close to the basilar membrane, whereas stromal cell density was decreased (**Fig. 2D**). In the 28d group, the cell-cavity surface with irregular margins contained a large number of small secretion bubbles. The interstitial edema was very observed, and stromal cells were enlarged with nuclei clearly showing a typical decidual-like stromal change (**Fig. 2E**). In the 31d group, a large number of leukocytes were infiltrated, and the endometrial tissue structure was disintegrated with erythrocyte leakage (**Fig. 2F**).

### Reticular fibers

**Figure 3:**
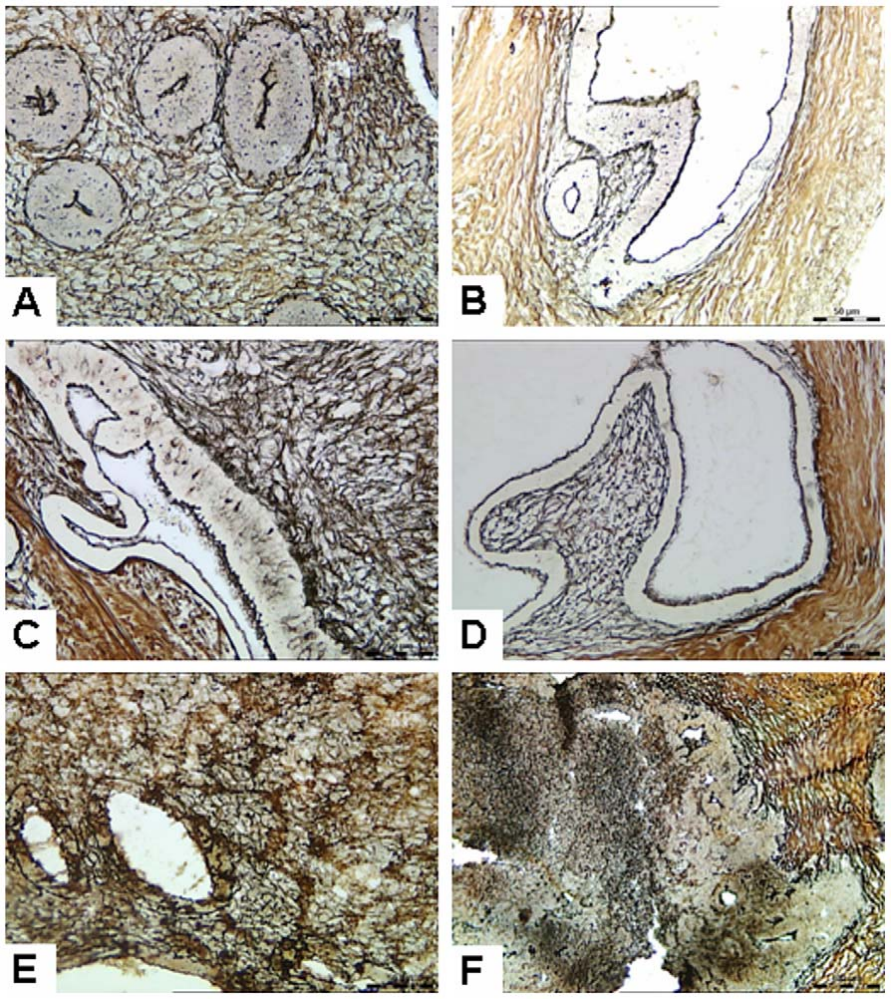
Reticular fiber staining of xenotransplanted human endometrial tissues. (**A**) Human endometrial tissue before transplantation. (**B**) Control group. (**C**) 14d group. (**D**) 21d group. (**E**) 28d group. In the interstitial region, black fiber wire could be seen clearly, and the mesh structure remained intact before transplantation, or in the control group, or in the 14, 21 and 28 group. (**F**) 31d group. Black mesh fibers were broken in some parts of the interstitial region, fiber mesh structure disappeared, and no black filaments were observed in some areas. Figure is from serial thin sections. Original magnification: 400×.

In the interstitial region, black fiber wire could be seen clearly, and the mesh structure remained intact before transplantation (**Fig. 3A**), or in the control group (**Fig. 3B**), and the 14, 21 and 28 group (**Fig. 3C, D, E**). In the 31d group, black mesh fibers were broken in some parts of the interstitial region, fiber mesh structure disappeared, and none of the black filaments were observed in some areas (**Fig. 3F**).

### Concentrations of estradiol (E_2_) and progesterone (P_4_) in serum

Concentrations of E_2_ and P4 in serum of SCID mice were measured at 7, 14, 21, 28 and 31 days after transplantation. The results were shown in **Table 2**. The E_2_-filled tubes implanted were 0.65 cm in length during week 1, 3 and 4, whereas they were changed into 1 cm during week 2. Therefore, the E_2_ serum concentration was increased and reached a maximum of 199.2±37.1 pg/ml at day 14, which could mimic the change of E_2_ level before ovulation in vivo. In the first two weeks when P_4_-filled tubes were not implanted, the serum concentration of P_4_ was reduced to a low level. Following insertion of the P_4_ implant, the serum concentration of P_4_ rose to 20.9±4.9 ng/ml (21d after transplantation), and 21.3±5.2 ng/ml (28d after transplantation). After P_4_ withdrawal, the serum P_4_ concentration decreased rapidly to 1.7±1.2 ng/ml, similar to the level before transplantation.

**Table 2:** Determination of serum concentrations of estradiol (E_2_) and progesterone (P_4_) (n = 6).

Time	E_2_ (pg/ml)	P_4_ (ng/ml)
7d	118.9±19.9	ND
14d	199.2±37.1	ND
21d	121.9±27.6	20.9±4.9
28d	73.2±13.5	21.3±5.2
31d	ND	1.7±1.2

ND: not determined.

### Expression of vimentin and cytokeratin

**Figure 4:**
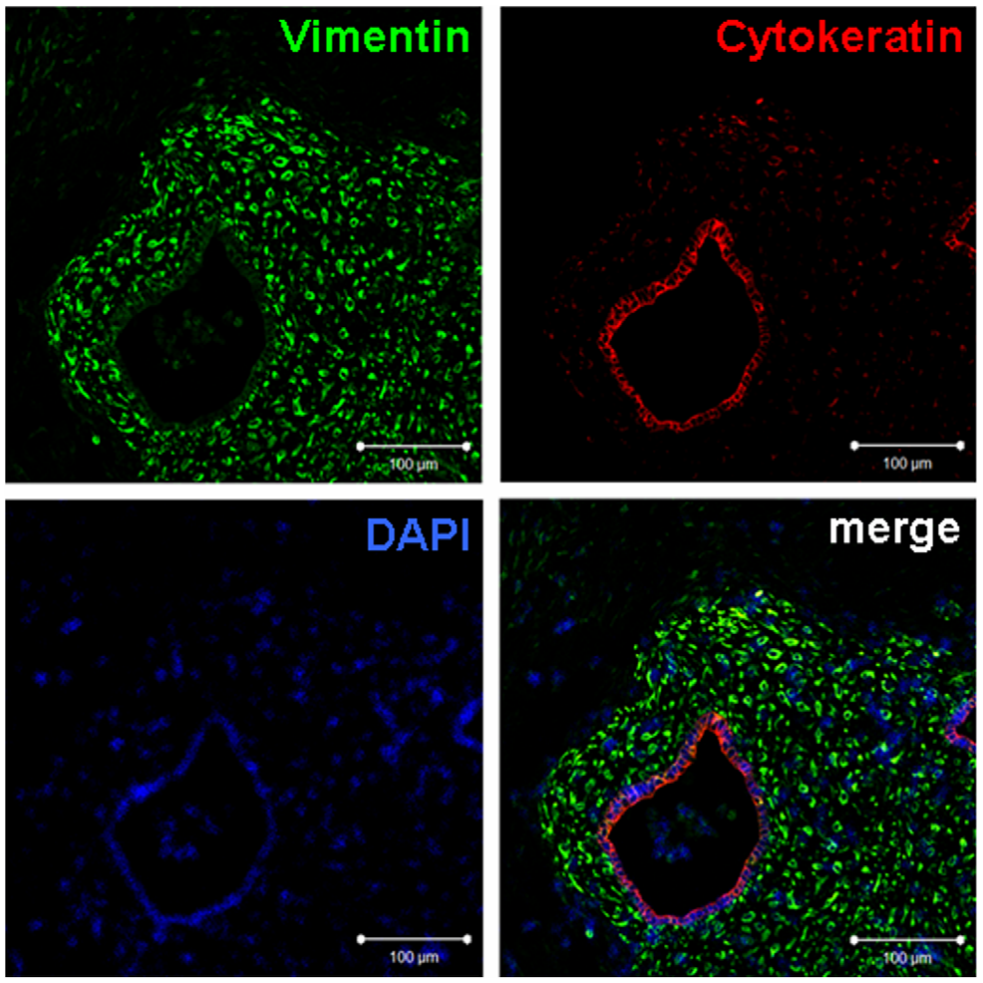
Immunofluorescence for human vimentin and cytokeratin after transplantation. The harvested tissues were detected by dual immunofluorescence for human vimentin and cytokeratin 28 days after transplantation. Stromal cells were specifically stained with vimentin (green), while epithelial cells were specifically stained with cytokeratin (red). Nuclei were visualized by DAPI (blue). Bars: 100 µm. Figure is from serial thin sections. Original magnification: 400×.

The anti-human vimentin and cytokeratin antibodies used in our study do not cross-react with mouse tissues, and can specifically label transplanted human tissues in mice. The green fluorescence, red fluorescence and blue fluorescence showed human stromal cells, human epithelial cells, and DAPI (Sigma, St. Louis, Missouri, USA)-labeled nuclei, respectively. As shown in **Fig. 4**, the areas outside the regions of green and red fluorescence were mouse tissues, while the green-fluorescence-positive and -negative regions were clearly demarcated. Fibrous connective tissues around the xenotransplanted tissues were green-fluorescence negative, indicating that they were mouse tissues, rather than human endometrial tissues.

### Leukocyte infiltration in human endometrium xenografts

**Figure 5:**
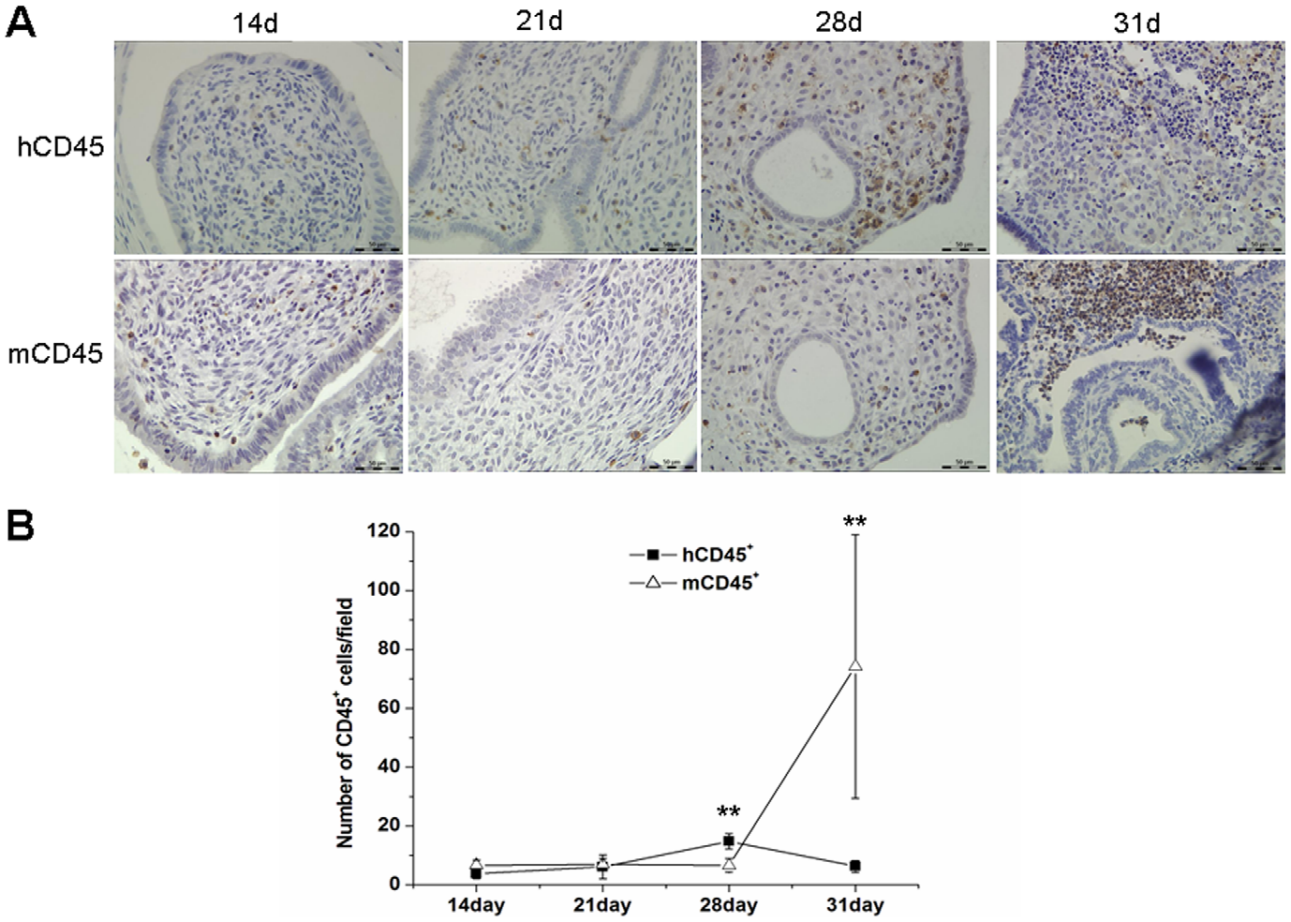
Immunohistochemistry detection of hCD45 and mCD45. (**A**) hCD45 and mCD45 staining from the 14d, 21d, 28d, and 31d groups. Original magnification: 400×. (**B**) hCD45^+^ and mCD45^+^ cell counts. Number of hCD45^+^ or mCD45^+^ cells was counted according to staining results. Statistical significance was assessed by the Student's *t*-test. **P<0.01.

A small number of leukocytes, derived from both human or mouse, were observed in xenografts of 14d and 21d group. In 28d group, the human-derived leukocytes (hCD45^+^) increased significantly, while the number of mouse-derived leukocytes (mCD45^+^) did not change significantly (**Fig. 5A**). However, in 31d group, hCD45^+^ cells decreased while the number of mCD45^+^ cells increased significantly (**Fig. 5A**).

Statistical analysis (**Fig. 5B**) showed that the number of hCD45^+^ cells in the 28d group was significantly higher than that of the 14d, 21d or 31d groups (*P*<0.01). However, the number of mCD45^+^ cells in the 31d group was significantly higher than that of the other groups (*P*<0.01).

### Detection of prolactin (PRL)

**Figure 6:**
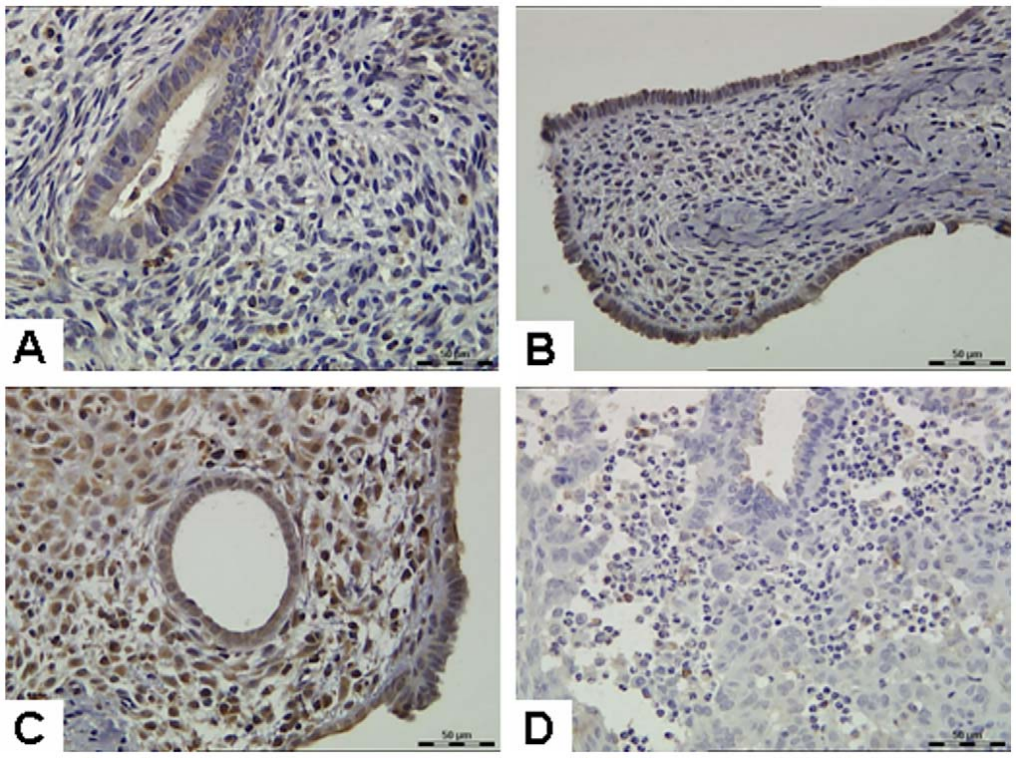
Expression of prolactin (PRL) protein. (**A**) 14d group; (**B**) 21d group; (**C**) 28d group; (**D**) 31d group. Original magnification: 400×.

In this study, we found that human stromal cells underwent decidual-like morphologic changes 28 days after human endometrium transplanted. To further confirm this finding, we measured levels of PRL, one of markers of decidualization. After 14 days of hormone treatment (14d group), PRL was mainly expressed in glandular epithelium, while stromal cells were weakly expressed (**Fig. 6A**). Compared with the 14d group, the 21d group had a higher PRL expression in the glandular epithelium, with no significant increase in the stromal cells (**Fig. 6B**). Glandular epithelial and stromal cells showed a strong PRL expression 28 days after treated with hormones (28d group) (**Fig. 6C**). In the 31d group, expression of PRL was not found in the glandular epithelium, with significantly reduced expression in the stromal cells (**Fig. 6D**). These immunohistochemistry results are consistent with the morphological results which showed that decidualization occurred in endometrial stromal cells in the 28d group.

### Expression of CD31 in human endometrial xenografts

**Figure 7:**
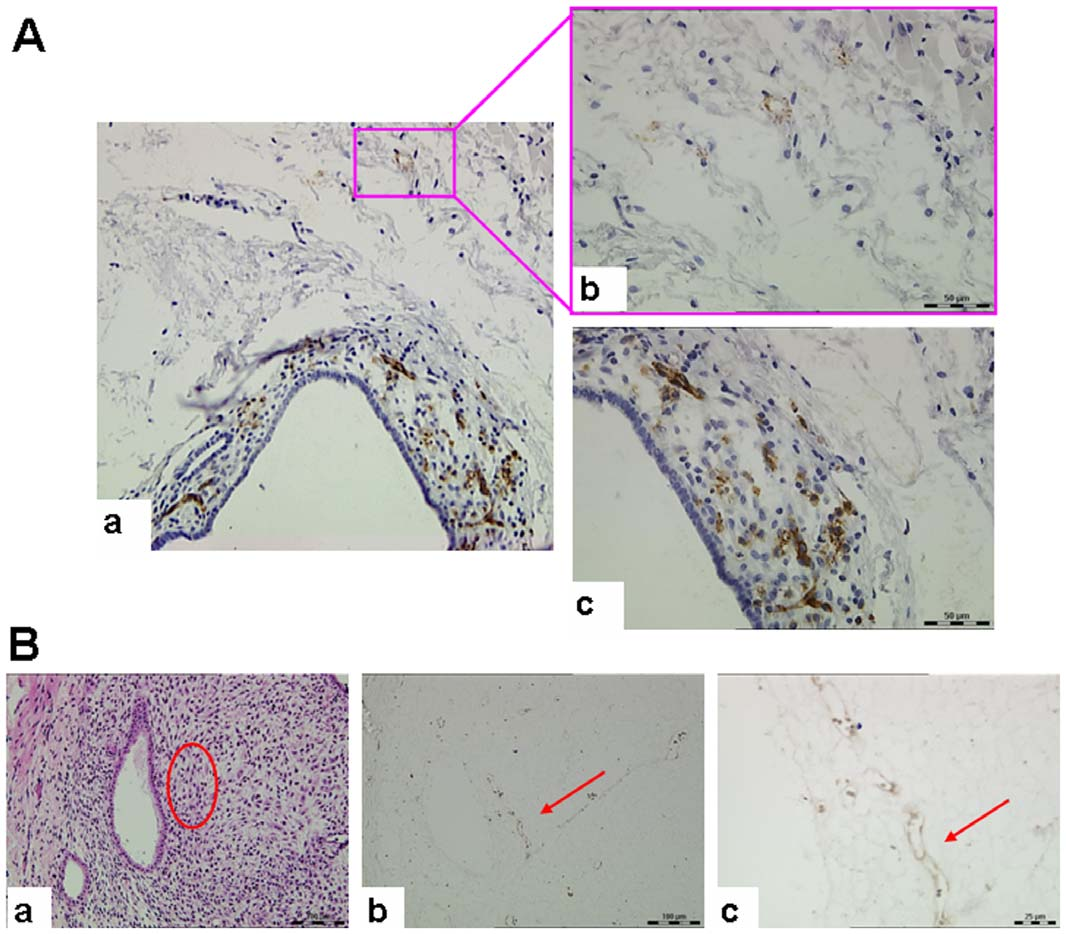
Human and mouse CD31 expression in xenotransplanted endometrial tissues. (**A**) hCD31 expression in endometrial tissues after transplantation. There were a large number of human blood vessels in transplanted tissues, while the mouse tissues surrounding transplanted tissues also have hCD31 expression. The image in the right (b, c) is a magnification of the image on the left (a). Original magnification: 400×. (**B**) mCD31 expression in endometrial tissues after transplantation. (a) and (b) are from serial thin sections. The mCD31^+^ blood vessels were indicated by red arrowhead in (b, c) which is also indicated by oval-shaped region in (a). (c) is a magnification of (b). Original magnification: 400×.

General observation showed that transplanted tissues were increased in volume, which indicated that the blood supply must be established between transplanted human tissues and host mouse tissues. In our study, a large number of human CD31^+^ cells were observed in transplanted tissues, suggesting that transplanted human tissues were rich in human blood vessels (**Fig. 7A c**). Mouse tissues around the transplanted human tissues also contained a small amount of human CD31^+^ signals, suggesting the growth of human blood vessels into the near mouse tissues or the infiltration of human endothelial cells (**Fig. 7A b**). We also found that the transplanted tissues contained mouse CD31^+^ cells forming duct-like structures (**Fig. 7B**), which indicated that transplanted tissue also contained mouse blood vessels.

### Expression and localization of MMP-1, 2 and 9

**Figure 8:**
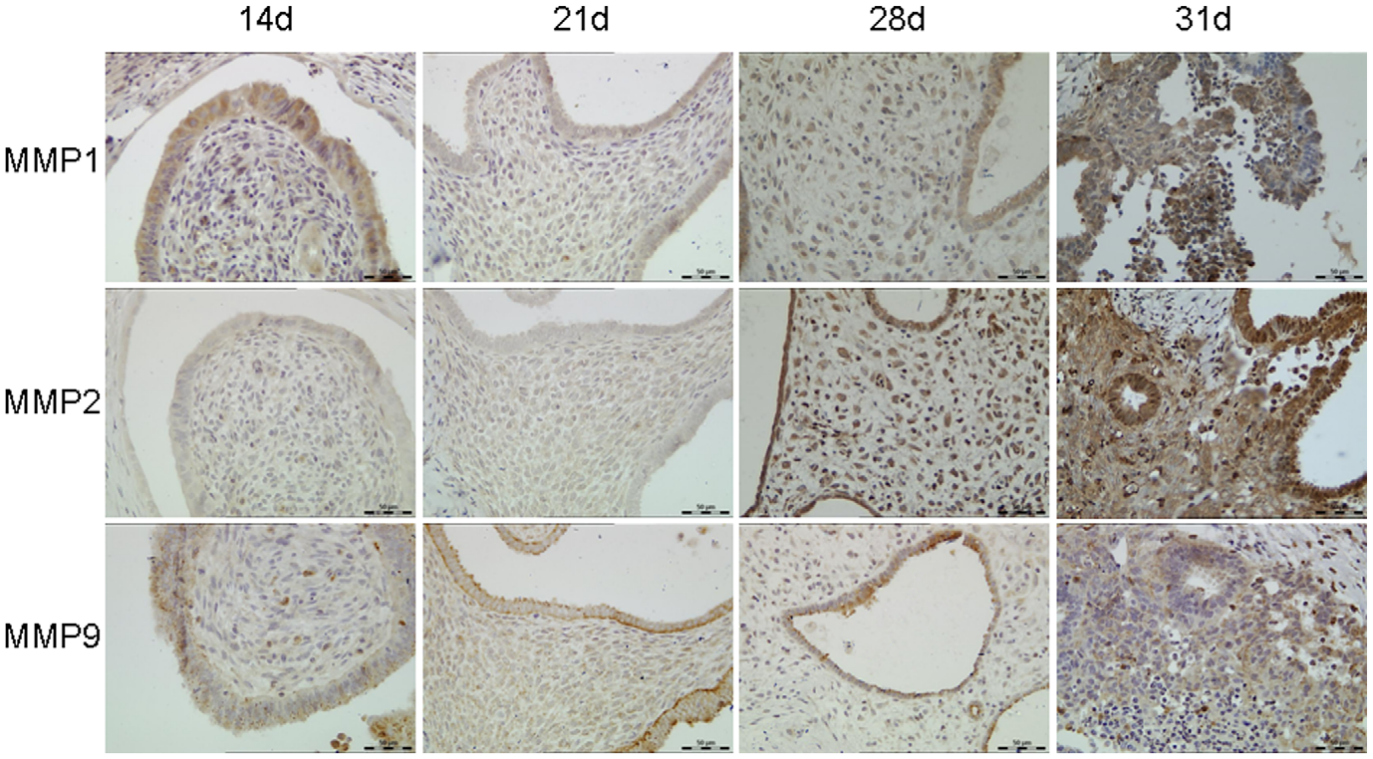
Immunohistochemistry of MMP-1, 2, and 9 in transplanted tissues. During the experiment stages, MMP-1, 2 and 9 had different changes in expression level. MMP-1 was expressed in the glandular epithelium and stroma in 14d, 21d and 28d group, and MMP-1 expression was increased in 31d group. MMP-2 was weakly expressed in transplanted tissues in 14d and 21d group, whereas expression in epithelial and stromal cells was significantly increased in 28d and 31d group. MMP-9 was weakly expressed in glandular epithelium and stromal cells, with no change in 14d, 21d and 28d group in expression level. However, MMP-9 expression was increased significantly in 31d group. Original magnification: 200×.

Immunohistochemistry results showed that MMP-1 was expressed in the glandular epithelium and stroma 14 days after tissue transplantation. There was no significant change for MMP-1 in the 21d and 28d groups, except in the 31d group in which MMP-1 expression in both glandular epithelial and stromal cells was increased. MMP-2 was weakly expressed in transplanted tissues on day 14 and 21, whereas expression in epithelial and stromal cells was significantly increased on day 28 and day 31. MMP-2 was mainly expressed in the collapsed areas of the xenografts. MMP-9 was weakly expressed in glandular epithelium and stromal cells, with no change in the 14d, 21d and 28d groups at protein level. However, MMP-9 expression was increased significantly in the 31d group (**Fig. 8**).

## Discussion

There are ethical and monetary considerations involved in primate animal models with limited resources. The tissues transplanted in human xenograft model can be grown and intervened in a well-controlled manner. In fact, human endometrial xenograft model is also suitable to evaluate drug effects on the menstrual cycle. In contrast to the other models such as cell culture system or mice, our model mimics the in vivo endocrine environment in which human menstruation occurs, by means of administration and withdrawal of exogenous human hormones in SCID mice, and remains human menstrual tissue characteristics and integrity. Here, based upon the results reported by Matsuura-Sawada *et al.* [12], we improved the method of hormone support by means of silastic implants that permit a slowly releasing of the hormones [2]. We detected and analyzed the leukocyte infiltration, vascularization and expression levels of MMP-1, 2 and 9 at indicated time points in our model. However, there are also limitations in our model, including a low recovery rate of xenografts and the difficulties of practice, which need to be improved in future studies.

Estradiol and progesterone play important roles in the proliferation and differentiation of endometrial cells. In this study, during the 14 days for 17β-E_2_ provided, there was proliferation of both epithelial and stromal cells. After P_4_ was provided, the epithelial cells contained a large number of secretory vesicles and glandular secretions. Exfoliating of epithelial cells was increased, and the stroma showed typical decidual-like changes which were confirmed by the detection of PRL. P_4_ withdrawal was followed by infiltration of a large number of leukocytes, tissue degeneration, and leakage of red blood cells. In response to the exogenous hormones, the transplanted human endometrial tissues showed the menstrual-like changes. These findings are consistent with those reported by Matsuura-Sawada *et al*. [12], [13]. Of note, the human tissues transplanted were enclosed completely by mouse connective tissues with the glandular openings and blocked luminal secretions, resulting in an increased luminal diameter, which is different from the normal endometrial tissues in vivo.

The counts and types of leukocytes in the endometrial stroma were changed during the menstrual cycle. Previous studies indicated that estrogen can increase the infiltration of leukocytes, whereas progesterone can antagonize this effect [14]–[16]. In our study, there were both human-derived leukocytes and mouse-derived leukocytes found in xenografts. The number of human leukocytes reached a peak in the 28d group, whereas 3 days after P_4_ withdrawal, the number of mouse leukocytes increased rapidly. It may be due to the recruitment of bone marrow-derived mouse leukocytes, and peripheral blood leukocytes of mouse origin may participate in the degeneration and tissues repair. These results suggested that human leukocytes were involved in human decidualization and cytokine secretion, whereas the mouse leukocytes were involved in tissue breakdown and repair.

It was previously believed that menstruation-generated leukocytes were from peripheral blood, however, the surface markers of uterine nature killer (uNK) cells from peripheral blood differ from those of uNK cells from the endometrium. This finding prompted researchers to reconsider the theory of leukocytes originating from the peripheral blood. In the 1970's, Dallenbach-Hellweg *et al*. [17] proposed that the leukocytes were differentiated from undifferentiated endometrial stromal cells. In 2005, using an allogeneic transplantation model, Matsuura-Sawada *et al*. [12] showed that uNK cells may originate from in situ proliferation. In our xenotransplantation model, the number of human leukocytes in transplanted tissue was increased, which indicated that leukocytes may originate from in situ proliferation. There are two possible explanations for the in situ proliferation of leukocytes: (1) precursors of leukocytes in the transplanted human endometrial tissue matrix can differentiate into leukocytes; (2) endometrial stromal cells can differentiate into leukocytes. The more direct evidence for this issue should be provided in future studies.

Endometrial blood vessels play an important role in the occurrence of menstruation. The presence of spiral arteries in the upper two-thirds of the functional layer was thought to be one of the characteristics of menstrual animals. However, despite the absence of spiral arteries in our study, the transplanted endometrial tissue also underwent disintegration, menstrual bleeding and other related events. This is consistent with Matsuura-Sawada R *et al*.'s report [12]. Moreover, we found that transplanted human endometrial tissues contained blood vessels of the host mouse. Masuda *et al*. [18] firstly proposed a human-host animal vascular chimera hypothesis based on an endometrial transplantation model. Recently, Alvarez Gonzalez *et al*. [19] also showed that the human-mouse chimeric vessels were present in human endometrial grafts. These studies, along with our histological results, convince us to assume a functional link of blood vessels between human and mice in our model.

The unique pattern of expression of MMPs during the various stages of the menstrual cycle indicates that MMPs play an important role in this process. The first step of collagen degradation is completed by MMP-1, followed by the initiating of the other MMPs expression [1]. Brenner *et al*. [20] found that MMP-1 mRNA expression increased after progesterone withdrawal in macaque menstrual cycle. Consistent with the above report, we also found that the expression of MMP-1 protein was increased in a similar manner after progesterone withdrawal in glandular epithelial and stromal cells. Meanwhile, our results suggested that MMP-2 may be associated with later stage of tissue repair. Using a mouse model, Kaitu'u *et al*. [21] detected an immune response between MMP-9 and neutrophils during the menstrual period, confirming the participation of MMP-9 in menstrual endometrial breakdown and repair. In our study, 3 days after progesterone withdrawal, MMP-9 expression was significantly increased, indicating that progesterone has an inhibitory effect on the expression of MMP-9.

In summary, we successfully established a human endometrial xenotransplantation model of menstruation. In this model, human endometrial xenograft tissues showed the menstrual-like changes responding to the exogenous hormone in SCID mice. A functional link of blood vessel between host mice and xenografts is concluded to be set up. MMP-1, 2 and 9 were shown to be involved in this process. Most importantly, using this model, we showed that leukocytes were originated from in situ proliferation in human xenografts and involved in the occurrence of menstruation. It is believed that this model will help to improve our understanding of growth, differentiation of the endometrium, and the underlying mechanism of menstruation.

## Materials and Methods

### Ethics statement

Female SCID mice (N = 45) aged 7 to 8 weeks were purchased from the Laboratory Animal Center, Academy of Military Medical Science (SCXK JING 2007-0001) and were housed under pathogen-free conditions in the barrier animal facility. All mice were conducted under approved guidelines of Laboratory Animal Center of Academy of Military Medical Sciences (SYXK JUN 2002-001). All procedures of the animal study were approved by the Ethics Committee of National Research Institute for Family Planning, China (Approval ID: 20100318).

Since all of the samples were anonymous without involving patients' curative effects in this study, the verbal informed consent was obtained from all patients. All procedures for informed consent and collecting human specimens were approved by the Ethics Committee of National Research Institute for Family Planning, China.

### Human endometrial tissues

From July 2008 to October 2009, human endometrial tissues were taken from 12 patients with myoma uteri or other benign gynecological diseases. Patients ranged in age from 32 to 47 years (average age 43 years). Samples were taken at the proliferative phase of the menstrual cycle. None of the patients received any hormonal treatment 3 months before surgery, whose menstrual cycles were regular. The menstrual phase of all the samples was determined by histologic examination.

### Preparation of silastic implants

Hormone-containing silastic implants were prepared as described in the literature [2], [22], [23]. Hollow plastic plugs (BioPortfolio, Dorset, UK) were inserted 0.25 cm into each end of a silastic tubing (inner diameter 1.57 mm, outer diameter 3.18 mm; Dow Corning, Midland, Michigan), making silastic tubing with hollow lengths of 0.65 cm, 1 cm, and 1.32 cm. One end of the hollow plastic plug was heat-sealed, and a 1 mL syringe was used to completely fill the tube with either 0.32 mg/ml 17β-E_2_ (Alfa Aesar, Heysham, UK) oil solution or 1 mg/ml P_4_ (Sigma-Aldrich, St Louis, Missouri, USA) oil suspension, respectively. Then the other end was heat-sealed. The E_2_-filled length in silastic implants was 0.65 cm or 1 cm. The P_4_-filled length in silastic implants was 1.32 cm. The filled silastic implants were rinsed twice with 95% ethanol and once with sterile water, left to airdry at room temperature, and packed in 1 ml sterile plastic tubes for storage. Prior to implantation, silastic implants were incubated overnight at 37°C in phosphate-buffered saline (PBS) containing 1% fetal bovine serum (FBS, GIBCO, Grand Island, NY, USA), pH 7.0.

### Transplantation of endometrial fragments

Under sterile conditions, human endometrial samples were immediately placed in cold Hank's solution to remove blood and cellular debris. After washing for three times, the samples were cut with scissors into tissue fragments of approximately 1 mm×1 mm×2 mm, and placed on ice. The ovarectomized SCID mice, which were allowed to recover for 2 weeks to eliminate the influence of the sex hormones of the animals, were anesthetized. Eight to ten fragments of human endometrial tissues were transplanted into the back subcutaneously of each mouse.

The silastic implants were inserted subcutaneously at the time of transplantation. The day when tissue fragments were implanted was counted as day 0 (0d). For a 28-day cycle, hormone implants were changed every week, according to the schedules (**Fig. 1A**). After tissue transplantation, animals were divided into 5 groups, with 9 mice in each group as follows: control group, a group not given hormones; 14d group, a group with E_2_ implants alone for the first 14 days (0.65 cm of 17β-E_2_-filled implant for the first 7 days, and 1 cm of 17β-E_2_ for the second 7 days); 21d group, a group with E_2_ implants for the first 14 days (as described in 14d group), and with 17β-E_2_ (0.65 cm) and P_4_ for the following 7 days; 28d group, a group with E_2_ alone for the first 14 days (as in 14d group), and with E_2_ (0.65 cm implant) and P_4_ for the next 14 days; 31d group, a group of 31 days in which hormones were provided same as those in 28d group but the implants were removed after 28 days and remained for another 3 days.

### Histological analysis

Fragments of the endometrial implanted tissues were removed and fixed for at least 24 hours with 4% paraformaldehyde or zinc fixative, dehydrated in gradient graduated alcohol, cleared in xylene, embedded in wax, and sliced into 4 µm serial sections. One of serial sections was stained with hematoxylin and eosin (H&E) for histological identification.

### Reticular fiber staining

Reticular fiber staining was performed as described previously [2], [24]. Briefly, 4 µm paraffin sections were de-waxed and dehydrated with conventional methods. The paraffin sections were sequentially treated with 1% potassium permanganate oxidation liquid (5 min), 2% oxalic acid (5 min), 2% ferric ammonium sulfate (5 min), ammonia silver solution (1.5 min), 10% formaldehyde solution (5 min), and ponceau-picric acid (2 min), with 1–5 min of wash in distilled water between each step. After excess dyes were washed away with distilled water, the section was directly dehydrated by ethanol for 15 minutes, cleared in xylene, and mounted with neutral gum.

### E_2_ and P_4_ assays

Samples of whole blood collected from the different experimental groups were placed at room temperature for 30 min, centrifuged at 1000 *g* for 15 min to separate serum, and the serum was then stored at −20°C. In this study, P_4_ was measured by radioimmunoassay (Progesterone Radioimmunoassay Kit, BNIBT, China), and E_2_ was measured by chemiluminescence (Estradiol Chemiluminescence Kit, BNIBT, China). The intra-assay coefficients of variation for E_2_ and P_4_ were less than 10% and 8%, respectively. The inter-assay coefficients of variation for E_2_ and P_4_ were less than 15% and 10%, respectively.

### Dual immunofluorescence of vimentin and cytokeratin

Paraffin sections at 4 μm were de-waxed and dehydrated with the conventional method. After antigen retrieval (**Table 3**), sections were blocked by goat serum for 40 min at room temperature. The primary antibody combination consisting of the rat anti-human vimentin and mouse anti-human cytokeratin was incubated with the sections overnight at 4°C. After three washes with PBS, the sections were incubated with FITC-conjugated goat anti-rat IgG antibodies (1∶50, Jackson ImmunoResearch Laboratories, West Grove, PA, USA) and TRITC-conjugated goat anti-mouse IgG antibodies (1∶50, Jackson ImmunoResearch Laboratories) for 45 min at room temperature. Nuclei were stained by DAPI for 5 min, mounted using 50% glycerol in PBS. The sections observed under an LSM 510 META laser confocal microscope (Zeiss, Carl Zeiss MicroImaging, Jena, Germany).

**Table 3:** Summary of the primary antibodies used in this study.

Antibody(clone number)	Source	Optimal dilution	Antigen retrieval
Human Vimentin(monoclonal: 280618)	R&D (Minneapolis, MN, USA)	1∶50	no retrieval
Human Cytokeratin(monoclonal: KL1)	AbD serotec (Oxford, UK)	1∶50	heat^a^, EDTA·Tris (pH9.0)
Human MMP-1(monoclonal: EP1247Y)	Epitomics (Burlingame, CA,USA)	1∶100	autoclave^b^, citric acid buffer
Human MMP-2(polyclonal)	Zhongshan Biotechnology (Beijing, China)	1∶150	autoclave^b^, citric acid buffer
Human MMP-9(polyclonal)	Zhongshan Biotechnology (Beijing, China)	1∶150	heat^a^, citric acid buffer
Human CD31(monoclonal: JC70A)	Dako (Glostrup, Denmark)	1∶40	heat^a^, EDTA (pH8.0)
Mouse CD31(monoclonal: MEC13.3)	Biolegend (San Diego, CA, USA)	1∶100	trypsin^c^
Human CD45(polyclonal)	Zhongshan Biotechnology (Beijing, China)	1∶200	heat^a^, citric acid buffer
Mouse CD45(monoclonal:30-F11)	BD Pharmingen(San Diego, CA, USA)	1∶150	heat^a^, citric acid buffer
Prolactin(monoclonal: C-17)	Santa Cruz (CA, USA)	1∶500	heat^a^, citric acid buffer

a heat: heat without boiling for 15 min.

b autoclave: heat in an autoclave for 2 min in citric acid buffer.

c trypsin: enzymatic digestion by trypsin for 10 min.

### Immunohistochemistry

Harvested endometrial tissues were cut into 4 µm paraffin sections using a slicer (Leica, RM2235, Germany). Paraffin sections were de-waxed and dehydrated with conventional methods. Each of antigens was retrieved according to the manufacturer's instructions (**Table 3**). Sections were incubated in 3% H_2_O_2_ at room temperature for 15 min. Nonspecific staining was blocked with 10% normal serum from the secondary antibody species. Sections were then incubated with the primary antibodies at 4°C overnight, respectively (**Table 3**), followed by HRP-labeled secondary antibodies (Zhongshan, Beijing, China) for 30 min at 37°C, with 3 washes in PBS between each step. DAB (3, 3′-diaminobenzidine) reagents were used to develop color, which is stopped when a light brown color was visible under the microscope. Nuclei were counterstained with hematoxylin for 15 min. PBS instead of primary antibodies was used as a negative control. Sections were dehydrated in gradient alcohol, cleared in xylene, and mounted with neutral gum.

### Histological assessment using digital imaging

CD45^+^ cells from the different experimental groups were counted by two independent observers, using a Leica LMD 6000 microscope (Leica, Wetzlar, Germany), and the average of the two observers' counts was taken as the value of the specimen. The Leica LMD image processing system (version 6.3.1) was used to define a regional area on the transplanted tissue. Results were expressed as the number of positive cells counted/10000 µm^2^.

### Statistical analyses

Hormone levels were expressed as mean ± standard deviation. A comparison between the counts of the human and mouse white blood CD45^+^ cells was performed using the Student paired *t*-test, while a comparison among different time points for human CD45^+^ cells or mouse CD45^+^ cells was performed using one-way ANOVA in SPSS11.0 statistical analysis software, respectively. A *P* value less than 0.05 was considered statistically significant.
