# Correction: Comprehensive Analysis of Leukocytes, Vascularization and Matrix Metalloproteinases in Human Menstrual Xenograft Model

**DOI:** 10.1371/annotation/77d64f53-b6fe-442d-9c29-7b749a99ddcf

**Published:** 2011-03-04

**Authors:** Yong Guo, Bin He, Xiangbo Xu, Jiedong Wang

There is an error in the funding section. The sentence "The Fundamental Research Funds for the Central Institutes (No: 2007GJSSJKA07)..." should be corrected to: "Central Public-interest Scientific Institution Basal Research Fund (Grant No.2009GJSSJKA02)..."

